# Optical fractal in cryogenic environments based on distributed feedback Bragg photonic crystals

**DOI:** 10.1371/journal.pone.0291863

**Published:** 2023-09-21

**Authors:** Miaomiao Zhao, Xiaoling Chen, Qianjin Liu, Jinrui Liu, Jun Liu, Yang Wang

**Affiliations:** 1 Laboratory of Optoelectronic Information and Intelligent Control, Hubei University of Science and Technology, Xianning, China; 2 School of Electronic and Information Engineering, Hubei University of Science and Technology, Xianning, China; 3 Xianning Senior High School, Xianning, China; 4 Hubei Guanchi Intelligent Science and Technology Co., LTD, Xianning, China; 5 School of Resource and Environmental Science & Engineering, Hubei University of Science and Technology, Xianning, China; Universiti Brunei Darussalam, BRUNEI DARUSSALAM

## Abstract

We studied the optical fractal effect of the one-dimensional distributed feedback Bragg photonic crystals formed by semiconductor GaAs and dielectric TiO2. Light wave is transmitted in the intermediate dielectric slab and reflected back by the periodic photonic crystals at both ends, forming multiple fractal resonance output. The transmission channels expand exponentially by thickening the bulk in a cryogenic environment. The quality factor of each fractal resonant state improves with a greater periodic number of crystals. Furthermore, central wave of resonance has a blue-shift as the external pressure increases, while the influence of environment temperature on the fractal resonance could be ignored. It is hoped that our study can highlight the potential of these findings for designing multi-channel communication filters in cryogenic environments.

## 1. Introduction

The idea of photonic crystals was first introduced by two scholars working independently in 1987. Yablonovitch [1] proposed using the complete photonic bandgap of photonic crystals to suppress spontaneous radiation of the medium, while John [2] utilized the bandgap characteristics of photonic crystals to randomly introduce dielectric materials with different refractive indices, realizing spatial localization of the optical field. Subsequently, photonic crystals became a field of great interest. Photonic crystal is an artificial dielectric structure with a spatially periodic distribution of dielectric constant [3–6], and it has a photonic bandgap. Photons with frequencies and energies in the bandgap cannot enter the inside of photonic crystal and are completely prohibited from existing inside of photonic crystal [7, 8]. This feature makes photonic crystals have significant application value in optoelectronics and optical communications [9–22]. The photonic crystal is composed of various materials such as semiconductors [17–19], ordinary dielectrics [20], metals [21] and various unconventional materials [22]. By controlling its structure and periodicity, precise control and modulation of optical waves can be achieved. In optical communication systems, multi-channel filters and wavelength division multiplexers are widely used [16, 23–36].

Periodic photonic crystals possess photonic bandgaps in their frequency spectra. Photonic crystals with defects or impurities can support defect modes, which are resonance modes [37–39]. The photonic crystal allows resonant light waves to pass through without being reflected when their wavelength matches the resonance condition. However, the defect mode is relatively singular and cannot support multi-wavelength resonances. Non-periodic and quasi-periodic photonic crystals possess multiple defects, which can support multi-wavelength resonant output. These defects are capable of generating several resonant modes in the frequency spectrum, which can support the transmission of different wavelengths of light. However, the frequency interval between adjacent resonant states is not uniform in the frequency spectrum of non-periodic and quasi-periodic photonic crystals. This non-uniformity results from the complex and irregular arrangement of defects. A photonic crystal made of ordinary dielectric material is highly susceptible to changes in temperature, which can significantly impact its refractive index. As a result, the center frequency of the channel supported by the photonic crystal can vary with temperature fluctuations. Once the traditional filter or wavelength division multiplexer is formed, the center frequency of its filter channel is difficult to control. The refractive index of semiconductor materials is less affected by temperature in the cryogenic environment, and its space size is easily affected by external pressure.

As the frequency spacing between adjacent resonant states under standing wave resonance conditions is uniform in the spectrum, we consider using a composite of semiconductor materials and dielectrics to form a fractal structure [40–42]. This fractal structure can satisfy the condition of standing wave resonance of optical waves during transmission, thereby generating optical fractal effects and multiple fractal resonances. This special optical effect can be used to achieve the function of multi-channel filtering or wavelength division multiplexing [43, 44]. In this work, we used the semiconductor material *GaAs* and the dielectric material *TiO*_*2*_ to compose a one-dimensional distributed feedback Bragg photonic crystal. The impacts of factors such as temperature, pressure, number of periods, and the number of dielectric layers on the optical fractal resonance were thoroughly studied using the transfer matrix method [45]. Overall, we discovered that this one-dimensional distributed feedback Bragg photonic crystal has significant potential for multi-channel communication filtering. Our findings also provide important references for designing more complex and efficient photonic crystals. The work is structured as follows. Section 2 shows the theoretical model. Section 3 presents and discuss the simulation results. Summary appears in section 4.

## 2. Theoretical model and numerical method

**Fig 1:**
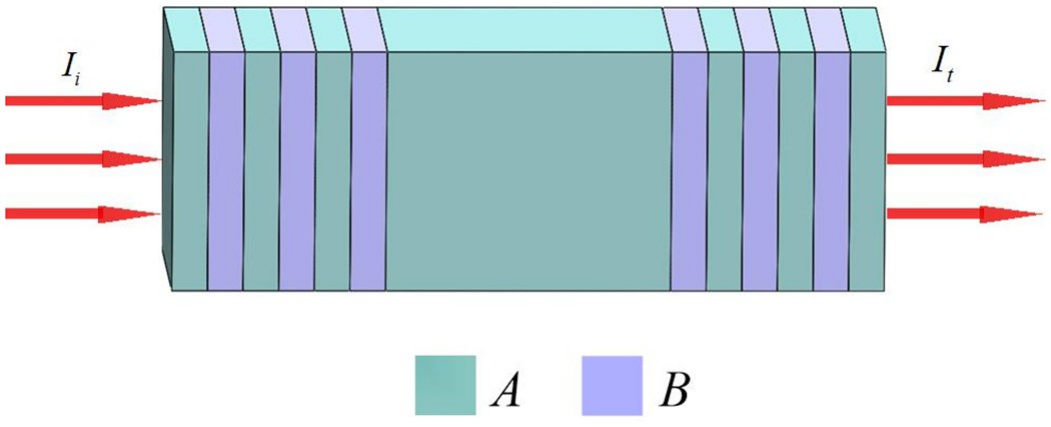
Schematic diagram of the one-dimensional distributed feedback Bragg photonic crystal structure (*AB*)^*N*^*C*(*BA*)^*N*^, with *N =* 3.

Fig 1 exhibits the one-dimensional distributed feedback Bragg photonic crystal configuration comprising two distinct dielectrics, *A* and *B*, systematically arranged in an alternating fashion, forming the periodic photonic crystals (*AB*)^*N*^ and (*BA*)^*N*^, the period number of the photonic crystal is denoted by *N*, where *N* (*N* = 1,2,3…) is any natural number. The above-mentioned two photonic crystals are, therefore, positioned at both terminations of the dielectric slab *C*, shaping a central symmetrical distribution structure, (*AB*)^*N*^*C*(*BA*)^*N*^, in a situation, where the structure of Fig 1 takes the form of *ABABABCBABABA*, given *N = 3*. The entire structure functions as a resonant cavity, where the dielectric material *C* acts as the cavity, and the two photonic crystals act as reflectors. The alternating arrangement of dielectrics in the two photonic crystals is equivalent to two Bragg gratings. In addition, their arrangement at both edges of the dielectric slab C results in the configuration of a distributed feedback Bragg photonic crystal.

The incident light is specified to be a transverse magnetic (TM) wave [39, 46], which enters the photonic crystal structure from the left, *I*_*i*_ and *I*_*t*_ are respectively referred to as the incident and transmitted lights.

One commonly used method for examining the transmission properties of photonic crystals is the transfer-matrix method (TMM) [34–36, 39]. The TMM simulation was performed using a homemade code in MATLAB software. We analyze a system containing multiple layers of materials (*n* layers) in order to calculate the transmittance. For each layer *i* in the system, we can use a matrix to represent the transmission properties of that layer.


$$M_{i}=\left[\begin{array}{cc} \text{cos}\;\delta_{i} & -\frac{i}{\eta_{i}}\text{sin}\;\delta_{i}\\ -i\eta_{i}\text{sin}\;\delta_{i} & \text{cos}\;\delta_{i} \end{array}\right].$$ (1)

Here,

$$\delta_{i}=\frac{2\pi n_{i}d_{i}\;\text{cos}\;\theta_{i}}{\lambda },$$ (2)

where *n*_*i*_ and *d*_*i*_ represent the refractive index and thickness for each layer (*i*) in the system, respectively. The variables λ and *θ*_*i*_ represent the wavelength and angle of incidence of the incoming light, respectively. When analyzing the transverse magnetic (TM) mode of an electromagnetic wave, the admittance (*η*_*i*_) of a given layer (*i*-th layer) can be expressed as

$$\eta_{i}=n_{i}\sqrt{\frac{\varepsilon_{0}}{\mu_{0}}}\frac{1}{\text{cos}\;\theta_{i}},$$ (3)

The equation involves several parameters, including the background admittance (*η*_0_), vacuum permittivity (*ε*_0_), vacuum permeability (*μ*_0_), and refractive indices (*n*_*a0*_ and *n*_*b0*_) of two different materials, *A* and *B*. These parameters are used to calculate the transmission matrix for each layer of a multilayer material structure. By multiplying these transmission matrices together, the overall transmission matrix for the whole structure can be obtained, that is

$$M=\prod_{i=1}^{n}M_{i}=\left[\begin{array}{cc} A^{\prime } & B^{\prime }\\ C^{\prime } & D^{\prime } \end{array}\right].$$ (4)

This approach can be used to analyze the behavior of light or other electromagnetic radiation as it passes through multilayer materials consisting of any number of layers.

The transmission coefficients of the photonic crystal can be calculated as

$$t=\frac{2\eta_{0}}{(A^{\prime }+B^{\prime }\eta_{0})\eta_{0}+C^{\prime }+D^{\prime }\eta_{0}}.$$ (5)

Then, the calculation of transmittance is to use the following formula,

$$T={\left|t\right|}^{2}.$$ (6)

In this study, we used the semiconductor material gallium arsenide (*GaAs*) as dielectric *A* and *C*, and titanium dioxide (*TiO*_*2*_) as dielectric *B*. Temperature and pressure make no influence on the photonic structure, which is only determined by the periodic number of Bragg photonic crystal and the defect layer C. However, hydrostatic pressure and environment temperature can change the refractive indices of materials.

The dielectric constant of Gallium Arsenide (GaAs) [47] is influenced by two factors: hydrostatic pressure (*P*) and temperature (*T*_*e*_). This relationship can be mathematically described as a function that takes into account both of these variables, that is [48]

$$\varepsilon_{B}\left(P,T_{e}\right)=12.74e^{-1.67\times 10^{3}P}e^{9.4\times 10^{-5}\left(T_{e}-75.6\right)},T_{e}<200K.$$ (7)

The thickness of *GaAs* varies with pressure (*P*) and can be described by

$$d_{\text{a}}\left(P\right)=d_{a\text{0}}\left[1-\left(S_{11}+2S_{12}\right)P\right],$$ (8)

where *d*_*a0*_ represents the original thickness at zero pressure. The elastic constants of *GaAs*, represented by the constants *S*_11_ and *S*_12_, have respective values of 1.16×10^−2^ GPa^−1^ and −3.7×10^−3^ GPa^−1^ which are used in the equation [28].

The relationship between the elastic constants of *TiO*_*2*_ and pressure can also affect the change in thickness.

$$d_{\text{b}}\left(P\right)=d_{b0}\left[1-\left(S_{11}+2S_{12}\right)P\right],$$ (9)

where *d*_*b0*_ is the original thickness at *P* = 0, and the elastic constants of *TiO*_*2*_ are *S*_11_ = 1.24×10^−2^ GPa^−1^ and *S*_12_ = −2.53×10^−3^ GPa^−1^, respectively [28].

The refractive index of *GaAs* at *T*_*e*_ = 0 K and *P* = 0 Gpa is *n*_*a0*_ = 3.5583. *TiO*_*2*_ displays consistent refractive index values within the terahertz (THz) frequency range. Neither variations in wavelength nor changes in temperature (ranging from 0 to 120 K) and pressure (up to 20 Gpa) have any significant impact on the refractive index [28]. At an incident wavelength of λ = 100 μm, the refractive index of *TiO*_*2*_ is measured to be *n*_*b0*_ = 5.81. In the given optical system, the input wavelength is defined as *λ*_0_ = 1.55 μm, and materials *A* and *B* have thickness values of 1/4 their respective optical wavelengths. Thus, the physical thickness of material *A* is given by *d*_*ao*_ = λ_0_/(4*n*_*a0*_) = 0.1089, while the physical thickness of material *B* is given by *d*_*bo*_ = λ_0_/(4*n*_*b0*_) = 0.0667 μm. The material of the dielectric slab *C* is also *GaAs*, and the original thickness of the dielectric slab *C* is set as *d*_*co*_ = 3^*M*^*d*_*ao*_ (*M* = 1,2,3…). This information can be used to calculate the behavior of light as it travels through these materials.

In contrast to previous studies on plasmonic metal-insulator-metal (MIM) filters with wide bandgaps and plasmonic ultra-wideband filters utilizing five symmetrically positioned half-circular resonators [49, 50], our research combinated semiconducting materials with dielectrics to form Bragg photonic crystals to realize optical fractal resonances, which arise in the photonic bandgap of light waves. Specifically, we examined a one-dimensional distributed feedback Bragg structure and evaluated the impact of various parameters on the transmission spectra. Our investigation considered factors such as layer thicknesses, the number of periods, as well as temperature and pressure conditions.

## 3. Numerical results and discussion

**Fig 2:**
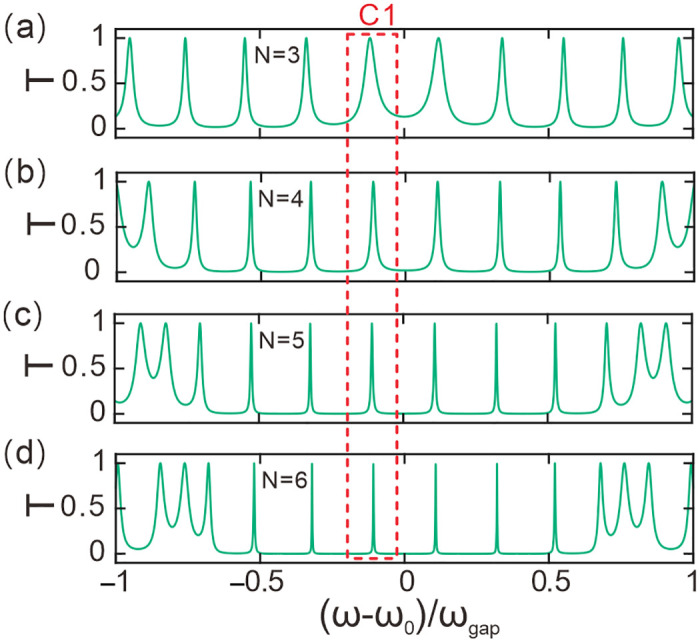
Transmittance spectra at different number of periods values. **(a-d)** The number of periods is *N =* 3, *N =* 4, *N =* 5, *N =* 6, respectively.

Fig 2(a) manifests the transmission spectrum varying with the normalized frequency for *N* = 3 with *T*_*e*_ = 10 K, *P* = 0 GPa. The thickness of dielectric slab *C* is initially set as *d*_*co*_ = 27*d*_*ao*_. The structure is *ABABABCBABABA* for *N* = 3. The notation *T* is the transmittance. (ω − ω_0_)/ω_gap_ is the normalized angular frequency. In the given scenario, the symbol ω is used to denote the angular frequency of light, which can be calculated using the equation ω = 2πc/λ. ω_0_ represents the central angular frequency and is calculated as ω_0_ = 2πc/λ_0_, where c is the speed of light. ω_gap_, on the other hand, is employed to describe the photonic band gap. We can calculate ω_gap_ using the formula ω_gap_ = 4ω_0_arcsin|(*n*_*a0*_−*n*_*b0*_)/(*n*_*a0*_+*n*_*b0*_)|2/π. From the figure, it can be seen that there are four independent sequence peaks in the interval (ω − ω_0_)/ω_gap_ = [−0.5, 0.5] of the normalized angular frequency. This suggests that photonic crystals have a special effect on the transmission of light waves in this interval. As a light wave passes through the dielectric slab *C*, it is reflected by the photonic crystals at both ends. This process is similar to the oscillation of a light wave in a resonant cavity. When the wavelength satisfies an integer multiple of half-waves, an optical fractal resonant state is formed. Only light waves that satisfy this condition will continue to resonate in the dielectric slab *C*. At this time, the light wave has a specific frequency and wavelength, and will be transmitted to form a communication channel. Therefore, we can consider this region as a result of the interaction between the light wave and the dielectric slab *C* and the two photonic crystals. The optical fractal resonances in the spectrum arising from the photonic bandgap are respectively coincident with their counterpart resonant states. Therefore, the material of slab C does not affect the main results of optical fractal resonances and their expansibility. This process uses the principle of resonance to achieve reflection and transmission, and by adjusting the parameters of the dielectric slab *C* to increase or decrease the frequencies of these channels, we can obtain more or fewer transmission channels.

Fig 2(b)–2(d) show the corresponding transmission spectra of the photonic crystals with *N* = 4, 5 and 6, respectively. The other parameters remain the same. It can be observed that in the interval (ω − ω_0_)/ω_gap_ = [−0.5, 0.5] of the normalized angular frequency, there are also four independent channels. Among these channels, the one closest to the center frequency (ω − ω_0_)/ω_gap_ = 0, labeled as C1, is highlighted with a dashed box for reference. As *N* increases, the optical fractal resonance peaks become increasingly narrower, indicating that the resonances become stronger. The stronger the resonance, the better the monochromaticity and wavelength selectivity of the channels. This structure is equivalent to a F-P resonant cavity, of which slab C is the cavity body and the periodic photonic crystals act as two reflection mirrors, so reflection is enhanced as the periodic number of photonic crystals increases. The resonance of optical fractal is also strengthened since the optical fractal resonance arises from the photonic bandgap. Resonance is directly proportional to the half width of resonant peak. Consequently, the bandwidth of the optical resonance decreaases as the periodic number. Therefore, we can conclude that the photonic crystals are able to produce high-quality transmission channels within the resonant frequency range, and the quality of the transmission channels can be improved by increasing the number of periods *N*. The wavelength selectivity and monochromaticity of the channels can also be enhanced by increasing the resonance strength, which proves the feasibility of using photonic crystals to design high-quality communication channels.

**Fig 3:**
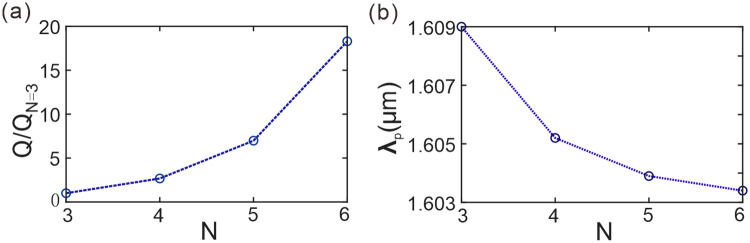
**(a)** The quality factor of the channel C1 varying with the number of periods. **(b)** The central wavelength of the channel C1 varying with the number of periods.

Fig 3(a) shows the relationship between the quality factor of channel C1 and the number of periods *N*. The quality factor *Q* is defined as the reciprocal of the half width of the resonant peak, where a narrower half-width results in a larger quality factor. Channel C1 has been labeled in Fig 2. The relative quality factor *Q/Q*_*N = 3*_ represents the relative value of the quality factor for different periods *N* with respect to *N* = 3. By observing Fig 3(a), it is evident that the relative quality factor exhibits an exponential increase with increasing period *N*. Higher quality factor of resonance approves of better monochromaticity of carrier channels or frequency selectivity of filters. This indicates that increasing the period *N* can enhance the resonance of the transmission mode, thereby improving the monochromaticity of the channel. This is highly beneficial for improving the quality and efficiency of information transmission in the channel.

Fig 3(b) depicts the trend of central wavelength variation in the photonic crystal channel C1 with respect to the number of periods. Here, *λ*_*p*_ denotes the central wavelength of the resonance mode in channel C1. The blue circles indicate the values of the central wavelength of the resonance mode in channel C1 at different numbers of periods. It can be observed that as the number of periods increases, the central wavelength of the resonance mode in channel C1 gradually decreases. This trend is due to the increase in the path length of light propagation inside the crystal with increasing period, which affects the propagation speed and hence results in the changes in the resonance wavelength. Therefore, this trend is of great importance and needs to be fully considered and evaluated in the design of photonic crystal devices.

**Fig 4:**
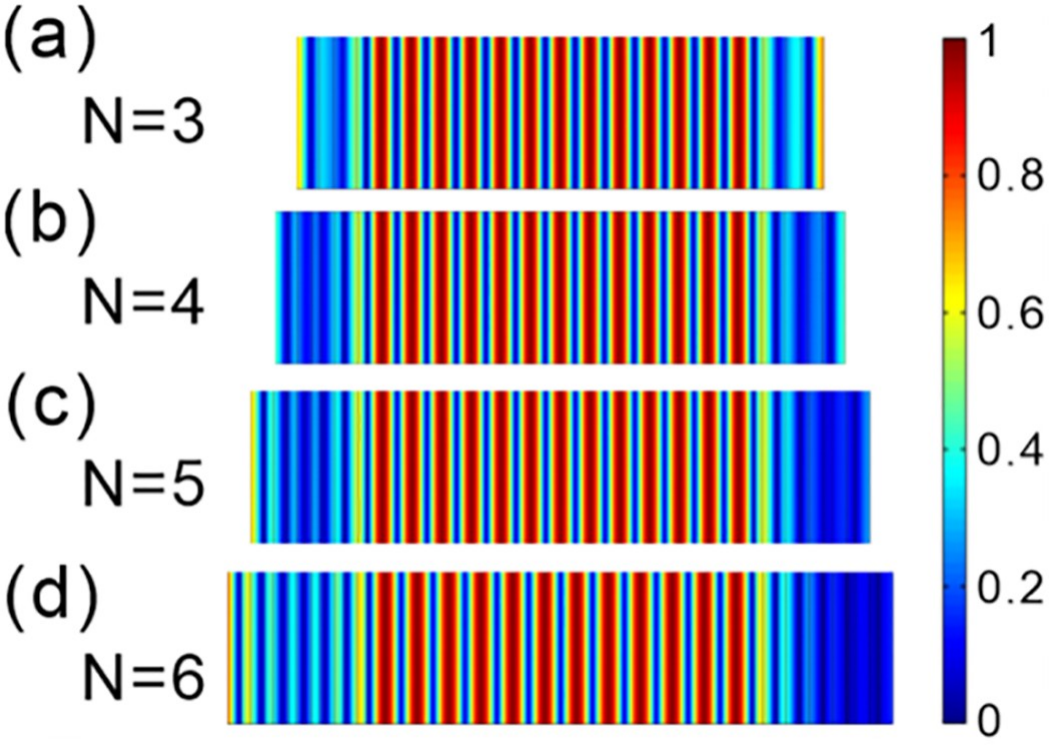
Electric field distribution of the optical fractal states in the transmission spectrum with different number of periods. **(a-d)** The number of periods is *N =* 3, *N =* 4, *N =* 5, *N =* 6, respectively.

Fig 4(a) depicts the normalized electric field intensity distribution of optical fractal states for *N* = 3 periods and an initial dielectric slab thickness of *d*_*co*_ = 27*d*_*ao*_, with the color bar representing the varying field strengths. Analysis of the image indicates that the electric field distribution of the resonance mode is approximatively symmetric to the central point. This phenomenon shows that the resonance mode has a significant localization effect on the electric field. When the wavelength satisfies the standing wave conditions, resonance occurs and the intervals between each resonance frequency are uniform, the resonant output is maximized.

Fig 4(b)–4(d) show the electric field distribution the photonic crystal at the number of periods *N* = 4, *N* = 5 and *N* = 6, respectively. As the light wave transmits in the structure, it also satisfies the standing wave resonance. When comparing the case where *N* = 3 to the current situation, it can be observed that the optical fractal resonance modes exhibit similar electric field distribution profiles. Moreover, despite the change made, the number of resonance modes remains unaltered. Therefore, for different values of *N*, the physical mechanism of the resonance transmission peak is the same. As the number of periods *N* increases, the intervals between the resonance frequencies is still uniform, and the electric field distribution of the transmission mode does not change. This optical fractal resonance phenomenon can be used in multi-channel communication to improve communication efficiency and capacity.

**Fig 5:**
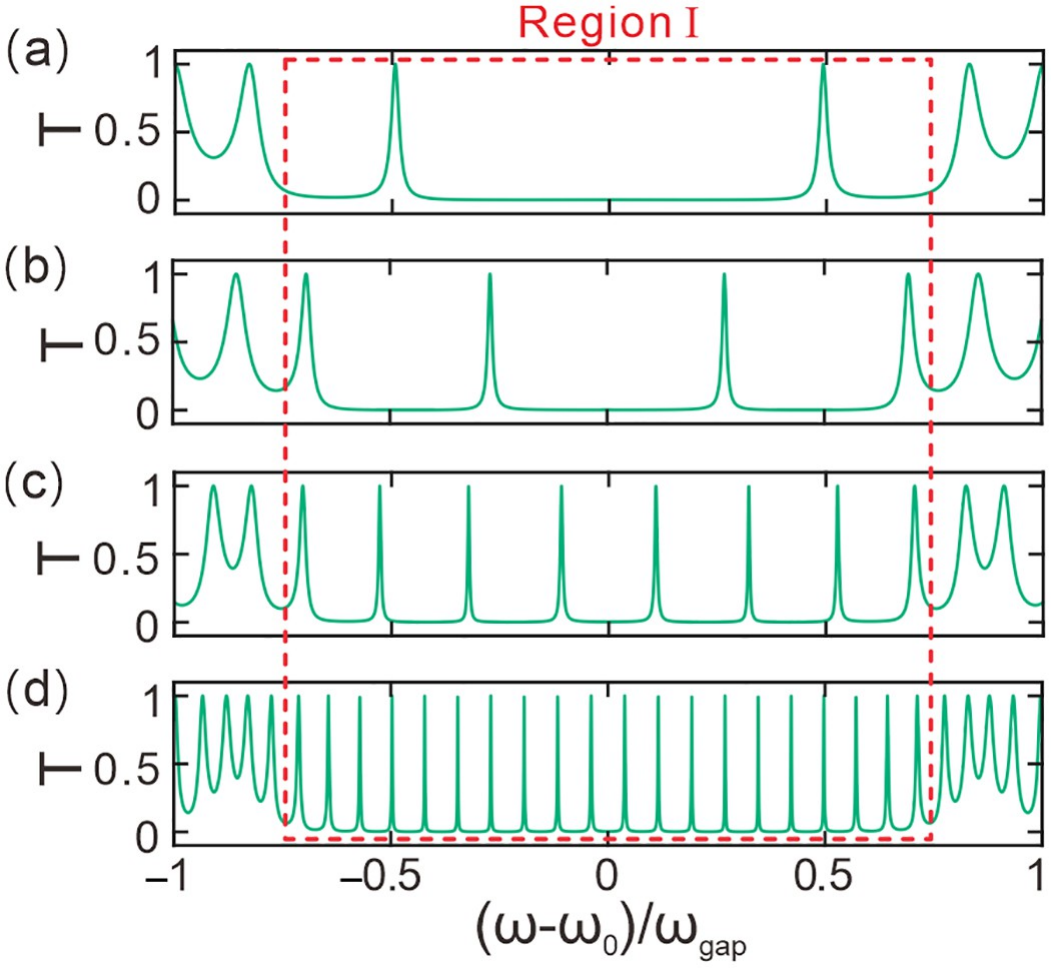
Transmittance spectra with different initial thickness of dielectric slab *C*. **(a-d)** The initial thickness is set to *d*_*co*_ = 3*d*_*ao*_, *d*_*co*_ = 9*d*_*ao*_, *d*_*co*_ = 27*d*_*ao*_, *d*_*co*_ = 81*d*_*ao*_, respectively.

Fig 5(a) shows the transmission spectrum of the photonic crystal with a dielectric slab *C* thickness of *d*_*co*_ = 3*d*_*ao*_ and a period number of *N* = 5, while keeping other parameters constant. It can be observed that the distribution of the resonance modes has symmetry.

Fig 5(b)–5(d) show the transmission spectra of the photonic crystal with dielectric slab *C* thicknesses of *d*_*co*_ = 9 *d*_*ao*_, 27 *d*_*ao*_ and 81 *d*_*ao*_, respectively, and the period number of *N* = 5, with other parameters kept constant. The distribution of the resonance modes also has symmetry, and compared with the case of *d*_*co*_ = 3*d*_*ao*_, the number of resonance modes increases significantly within the normalized frequency range of (ω − ω_0_)/ω_gap_ = [−1, 1], as the thickness of dielectric slab *C* increases. This indicates that adjusting the thickness of the dielectric slab *C* is an effective means of controlling the number of resonant modes. Therefore, in applications such as multi-channel communication, increasing the thickness of the dielectric slab *C* can be used to expand the number of channels.

**Fig 6:**
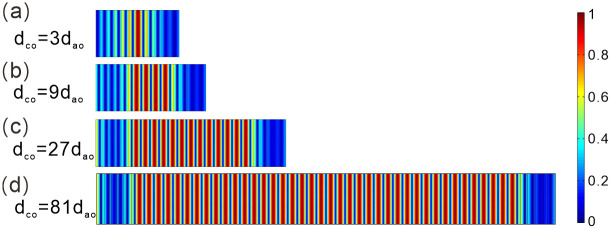
Electric field distribution of the optical fractal states in the transmission spectrum with different initial thickness of dielectric slab *C*. **(a-d)** The initial thickness is set to *d*_*co*_ = 3*d*_*ao*_, *d*_*co*_ = 9*d*_*ao*_, *d*_*co*_ = 27*d*_*ao*_, *d*_*co*_ = 81*d*_*ao*_, respectively.

Fig 6(a) shows the electric field intensity distribution of optical fractal states for *N* = 5 periods and an initial dielectric slab thickness of *d*_*co*_ = 3*d*_*ao*_, while keeping other parameters constant. The image displays a transmission mode whose electric field intensity distribution is approximatively symmetrical. The electric field largely inhabits the structure’s central regions, showcasing the highest resonant output. The resonance frequencies are uniformly distributed between each interval. At this time, the electric field intensity distribution of the transmission mode is relatively narrow.

Fig 6(b)–6(d) show the electric field intensity distribution of optical fractal states for the thickness of the dielectric slab *C* is *d*_*co*_ = 9 *d*_*ao*_, 27 *d*_*ao*_ and 81 *d*_*ao*_, respectively. Compared with the case of *d*_*co*_ = 3*d*_*ao*_, the distribution profile of the resonance modes is similar. Under the standing wave resonance condition, the intervals between each resonance frequency is still uniform. By observing the images, it can be clearly seen that as the thickness of the dielectric slab *C* increases, the electric field intensity distribution of the transmission mode becomes wider and the number of resonance modes increases. Therefore, by adjusting the thickness of the dielectric, we can control the resonant phenomenon and achieve the desired optical performance.

Table 1 shows the number of channels in the transmission spectrum within region I. Region I refers to the spectral band within the dashed box in Fig 5, i.e., the interval (ω − ω_0_)/ω_gap_ = [−0.743, 0.743]. It can be seen from the table that, as the thickness of the dielectric slab *C* increases, the number of wavelengths that satisfy resonance conditions gradually increases, and the number of channels in region I shows a geometric progression increase.

**Table 1:** Number of channels with different thickness of dielectric slab *C*.

Thickness of dielectric slab C	Number of channels in region I
*d*_*co*_ = 3*d*_*ao*_	2
*d*_*co*_ = 9*d*_*ao*_	4
*d*_*co*_ = 27*d*_*ao*_	8
*d*_*co*_ = 81*d*_*ao*_	20

**Fig 7:**
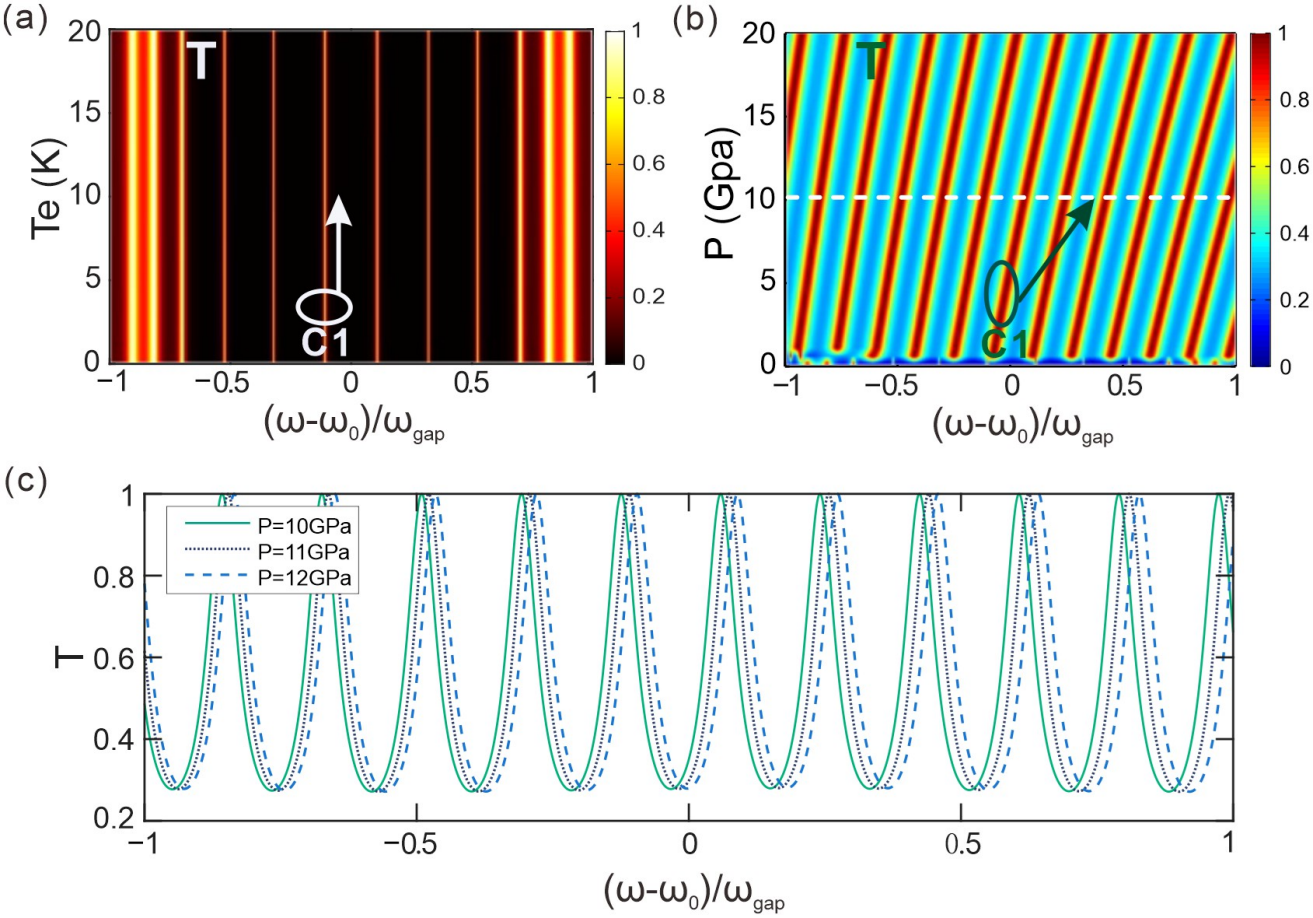
**(a)** The transmittance in the parameter space composed of temperature and normalized frequency. **(b)** The transmittance in the parameter space composed of pressure and normalized frequency. **(c)** The transmittance spectra corresponding to the external static pressures of *P* = 10 GPa, 11 GPa, and 12 GPa, respectively.

Fig 7(a) displays the transmittance of light waves in the parameter space of temperature and normalized frequency. Setting the static pressure to *P* = 0 GPa, the number of periods to *N* = 5, and the thickness of the intermediate dielectric plate *C* to *d*_*co*_ = 27*d*_*ao*_, with all other parameters remaining constant. Modifying the environmental temperature and the refractive indices of materials for *A*, *B* and *C* may vary ever so slightly. As observed from the graph, with a change in environmental temperature, the central frequency of the light frequency through channel C1 remains approximately constant, and other channels have similar characteristics. This implies that, within a certain range, a change in environmental temperature may have a negligible influence on the transmittance while maintaining the stability of channel transmission due to the consistent central frequency. Therefore, based on this property, we can design and optimize corresponding optical devices to cater to distinct environmental requirements and demands.

Fig 7(b) shows the transmittance of light waves in the parameter space constituted by pressure and normalized frequency. In this system, the environmental temperature is maintained at *T*_*e*_ = 10 K, the number of periods is *N* = 5, and the thickness of dielectric slab *C* is *d*_*co*_ = 27*d*_*ao*_, with other parameters remaining unchanged. As the external static pressure changes, the refractive indices of the dielectric films *A*, *B* and *C* are affected, thus altering the resonant condition for the incident wavelength of the light wave. As seen from Fig 7(b), with an increase in the external static pressure, the center frequency of channel C1 shifts towards the higher frequency direction, while similar phenomena are observed for other channels. Therefore, by adjusting the external static pressure, the center frequency of each channel can be flexibly controlled.

Fig 7(c) shows the transmitted light spectra corresponding to external static pressures of *P* = 10 GPa, 11 GPa and 12 GPa, respectively. All other parameters remain consistent with Fig 7(a). At *P* = 10 GPa, the transmittance corresponds to the dashed line position in the parameter space of Fig 7(a). As shown in Fig 7(c), with the external static pressure increases, channel C1 shifts to the right, i.e., towards the high-frequency direction. At *P* = 10 GPa, the central frequency of channel C1 is (ω − ω_0_)/ω_gap_ = − 0.1243; at *P* = 11 GPa, the central frequency of channel C1 is (ω − ω_0_)/ω_gap_ = − 0.1097; at *P* = 12 GPa, the central frequency of channel C1 is (ω − ω_0_)/ω_gap_ = − 0.0950. Consequently, the center frequency of each channel is mainly regulated by the external static pressure and less influenced by the environmental temperature in a low-temperature environment. The system can be tuned to the required central frequency by suitably adjusting the static pressure, with temperature playing a secondary role in this process.

## 4. Summary

We conducted research on the optical fractal effect of one-dimensional distributed feedback Bragg photonic crystals composed of *GaAs* semiconductor and *TiO2* dielectric. The multiple fractal resonance output forms in this structure, which can be used for multi-channel communication. The transmission channels expand exponentially as the thickness of the dielectric slab increases. The quality factor of each fractal resonant state improves with the number of periods of the fractal structure increases. Additionally, the central frequencies of each channel are largely unaffected by environmental temperature but can be adjusted using external static pressure. As the external pressure increases, the central frequencies of the channels shift towards higher frequencies. In contrast to previous studies [40–42], under the optical fractal resonance condition, the frequency spacing between two adjacent resonant states is uniform in our work. This uniform channel spacing provides efficient optical signal transmission and multiplexing capabilities, enabling photonic crystals to have extensive potential applications in optical communication, sensing, and information processing.

## Supporting information

S1 Data (ZIP)
